# A computer vision model for automated kidney stone segmentation and evaluation of its performance vs surgeons

**DOI:** 10.1111/bju.70001

**Published:** 2025-09-25

**Authors:** Daiwei Lu, Ekamjit S. Deol, Tatsuki Koyama, Ipek Oguz, Nicholas L. Kavoussi

**Affiliations:** ^1^ Department of Computer Science Vanderbilt University Nashville TN USA; ^2^ Department of Biostatistics Vanderbilt University Nashville TN USA; ^3^ Department of Urology Vanderbilt University Medical Center Nashville TN USA; ^4^ Saint Louis University School of Medicine St. Louis MO USA

**Keywords:** kidney stone disease, artificial intelligence, machine learning, computer vision, segmentation, computer‐assisted Surgery

## Abstract

**Objectives:**

To develop a computer vision model that segments stones to improve visualisation during ureteroscopy (URS) and to compare model performance to that of experts.

**Materials and Methods:**

We collected 136 videos of URS for intrarenal kidney stone treatment. Frames were extracted at 3 frames per second (FPS) and manually annotated. The video dataset was split into training (75%), validation (5%) and testing (20%) subsets. Model performance was evaluated for stone localisation, laser ablation, and final evaluation of remaining fragments based on area under the receiver‐operating curve, binary cross‐entropy loss and Dice similarity coefficient (DSC). Model performance was compared to the manual annotations of five board‐certified urologists through pairwise comparison of frame‐by‐frame segmentation accuracy.

**Results:**

The final dataset consisted of 21 718 frames from 38 fibreoptic and 98 digital videos. Overall, the model showed excellent performance: DSC 0.97 (interquartile range [IQR] 0.91, 0.99) and could segment at 30 FPS. Performance was similar for both fibreoptic (0.97 [IQR 0.91, 0.99]) and digital scopes (0.97 [IQR 0.92, 0.99]). Additionally, the model demonstrated good performance during stone localisation (0.98 [IQR 0.93, 0.99]) and stone laser ablation (0.96 [IQR 0.89, 0.97]), with slightly worse performance during evaluation of residual fragments (0.91 [IQR 0.50, 0.97]). Model performance was comparable to the five expert surgeons overall. In a head‐to‐head comparison, the model significantly outperformed three of the five experts and performed similarly to the other two.

**Conclusion:**

The computer vision model demonstrates good performance for task‐specific stone segmentation evaluation during URS. The segmentation performance of the model was similar to the segmentation performance of expert surgeons, demonstrating the feasibility of its real‐time intra‐operative utilisation.

**Abbreviations**
AUC‐ROC: area under the receiver‐operating characteristic curve
BCE: binary cross‐entropy
DSC: Dice similarity coefficient
FPS: frames per second
IQR: interquartile range
URS: ureteroscopy

## Introduction

Ureteroscopy (URS) with laser lithotripsy is the most commonly performed surgery for kidney stones [1]; however, postoperative residual stone fragments may be present in up to half of cases on postoperative imaging [2, 3]. This is partially because diminished endoscopic visibility during the procedure leads to missed stones or incomplete treatment. Specifically, ureteroscopes are limited by their narrow field of view, which is easily compromised by intra‐operative factors such as debris, bleeding, or turbid irrigation fluid [4]. These visibility challenges significantly hinder the detection and tracking of stone fragments, and impact complete stone treatment during URS.

We have previously demonstrated the feasibility of integrating computer vision models for the intra‐operative segmentation of kidney stones during URS [5, 6]. These types of models perform well in automatically segmenting kidney stones in real time, and could alleviate the visibility limitations of current scopes [5, 6, 7]. However, the small, homogenous datasets typically used for training computer vision models prevent the immediate application of a robust segmentation algorithm capable of consistent and reliable performance across varied clinical cases. For example, to our knowledge, no previous model has evaluated segmentation performance during the different surgical tasks of URS (stone localisation, stone laser ablation, and evaluation of residual stone fragments). Furthermore, to our knowledge, model performance has never been compared to the performance of expert surgeons. It is important to demonstrate that computer vision models can achieve stone segmentation performance comparable to that of experts to ensure their safe implementation in the operating room.

In this study, we aimed to leverage a large URS video dataset to enhance the robustness of a deep learning computer vision model for segmentation of kidney stones and to evaluate its performance during the distinct surgical tasks of URS. We further compared the model's performance against manual segmentation conducted by five board‐certified urologists.

## Methods

### Dataset Collection and Annotation

Following institutional review board approval and waiver of informed consent under the protocol, 142 *in vivo* URS videos of stone treatment were collected. The videos were obtained from both digital and fibreoptic scopes (Karl Storz Flex X^c^ and Flex X^2S^) and recorded via an Image1 S Connect system. The surgeries were performed by five different fellowship‐trained endourologists. Operative videos were manually reviewed for quality. Six of the videos were excluded due to artificial distortions which precluded visual analysis. Each of the remaining 136 videos was divided and categorised into three separate surgical tasks performed in URS: (i) stone localisation; (ii) stone laser ablation; and (iii) evaluation of residual stone fragments (Table S1). Stone localisation included initial identification and basket manipulation of kidney stones. Stone laser ablation included active stone ablation of kidney stones. Evaluation of residual stone fragments encompassed visualisation of stone fragments (i.e., dust) left in the kidney at the conclusion of a case. Surgical technique (i.e., use of an access sheath, and scope type) was left to the discretion of the surgeon for each case.

Individual video frames were extracted from the surgical videos at 3 frames per second (FPS). Under the guidance of a fellowship‐trained endourologist, each frame (*N* = 21 718) was manually annotated by two students to identify kidney stones from blood, debris and background structures. Each frame was visually verified by the fellowship‐trained endourologist who compared all frames to the URS videos to ensure accurate segmentation. This established the ground‐truth dataset of annotated frames for model training and validation. Manual annotation was performed on the Computer Vision Annotation Tool (CVAT) platform (CVAT.ai, Palo Alto, CA, USA).

In the 136 videos, stone localisation, stone laser ablation, and residual fragment evaluation were seen in 67, 92 and 24 of the videos, respectively. Digital scopes and fibreoptic scopes were used for 98 and 38 videos, respectively. The mean video duration was 53 s (sd 51 s).

### Model Development and Evaluation

To select an optimal model, we compared three modern baseline convolutional neural network architectures (i.e., U‐Net, U‐Net++, and UNeXt) [8, 9, 10]. These are common architectures for computer vision‐based tasks, with robust and well characterised performance. Additionally, these architectures are relatively compact (e.g., compared to transformer‐based architectures), enabling deployment on modest graphic processing units such as those found in modern laptops. The videos were divided into training, validation and testing sets (75%/5%/20%), with 102 (16 639 frames), 8 (1120 frames) and 26 (3959 frames) in each set, respectively. The models used the same training, validation and testing sets of the annotated frames for a fair comparison. We used publicly available implementations of U‐Net and U‐Net++ found at (https://github.com/qubvel‐org/segmentation_models.pytorch). We used the implementation of UNeXt available at (https://github.com/jeya‐maria‐jose/UneXt‐pytorch). For training, we used a standard iterative framework, involving forward propagation and loss computation, followed by back propagation with routine validation. To optimise model performance, a comparative evaluation of key hyperparameters – learning rate, batch size, number of epochs, and scaling factor – was conducted for each model. Model architectures were compared using three metrics: the Dice similarity coefficient (DSC), average area under the receiver‐operating characteristic curve (AUC‐ROC) of pixel‐based stone segmentation across all annotated frames for each task, and binary cross‐entropy (BCE) loss function. The U‐Net model demonstrated the highest DSC score performance, with comparable AUC‐ROC and BCE, and was selected for subsequent analyses of performance (Table S2). The U‐Net model's performance for stone segmentation based on scope type and each surgical task was evaluated using DSC.

### Model Comparison to Experts

To validate the performance of the computer vision model, we recruited five board‐certified urologists to manually annotate the same set of 200 frames randomly selected from the video dataset, ensuring the same proportion of each surgical task as the overall dataset (i.e., stone localisation, stone laser ablation, and residual fragment evaluation shown in 42%, 53%, and 5% of frames, respectively). We compared the segmentation performance of the model to the annotations of the five experts based both on the detection accuracy of stone presence and the level of segmentation agreement with the ground‐truth using the DSC. For each expert and the model, the accuracy of binary presence or absence of stone presence were estimated and reported with a 95% CI. For the frames with a stone present, the DSC was calculated for each expert and the model, and the median and quartiles were reported. Wilcoxon signed‐rank sum tests were used to determine if the DSC was significantly different between the model and each expert. To compare the model's performance to each expert, the proportion of frames where the model segmented stones more correctly (‘win’) was recorded, and the proportion of such model wins was estimated and reported with a 95% CI. All findings are reported via the Standardized Reporting of Machine Learning Applications in Urology framework to enhance the reproducibility, comparability and interpretability of our results [11]. Our models are available for public use and validation (see link).

## Results

### Model Performance

**Fig. 1:**
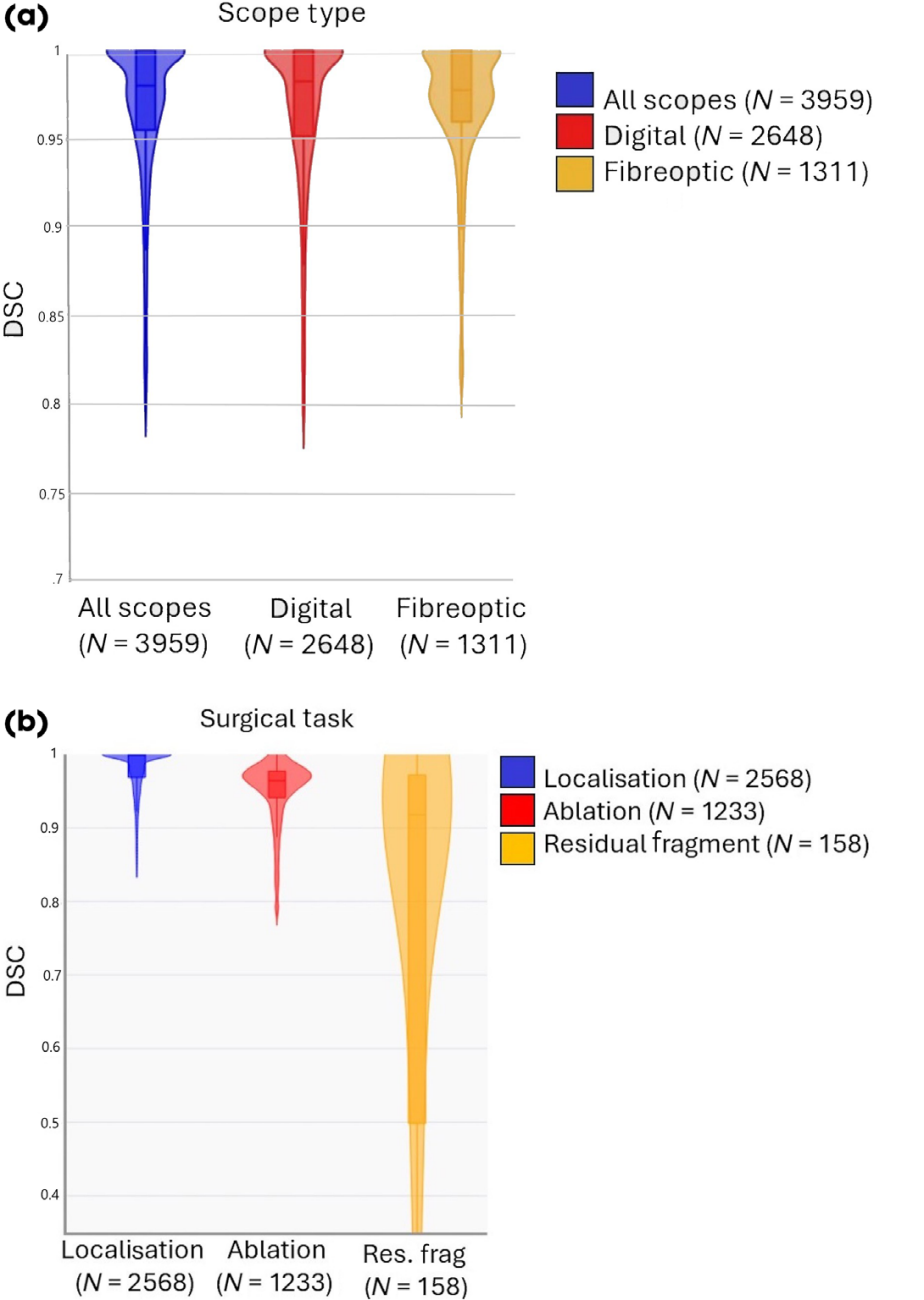
Comparison of median Dice similarity coefficients (DSCs) from final U‐Net model to demonstrate performance based on (**a**) ureteroscope type and (**b**) surgical task. Outliers are excluded.

**Fig. 2:**
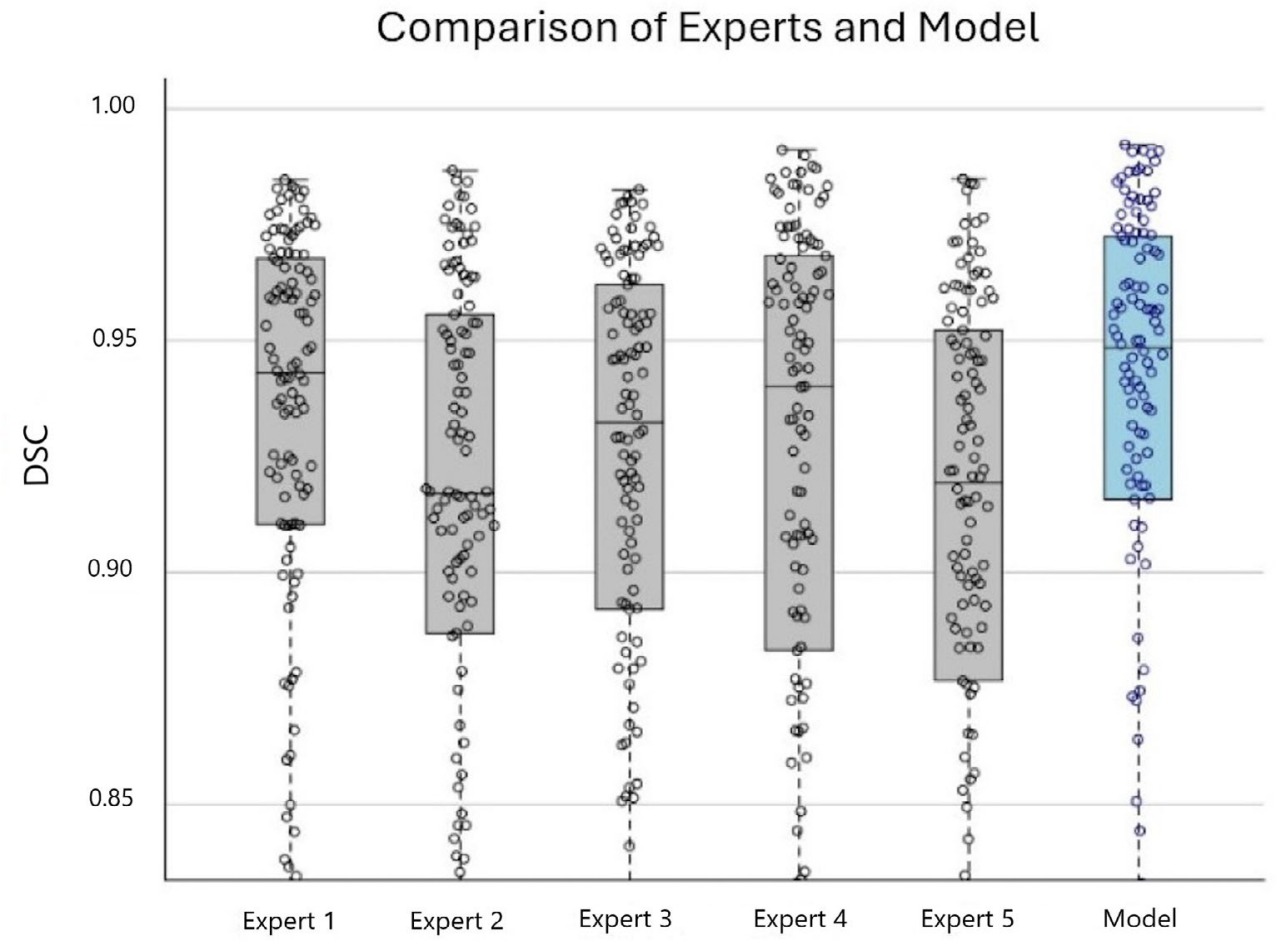
Box plot comparison of median Dice similarity coefficient (DSC) values among the model and the five experts.

The model demonstrated good performance overall for stone segmentation (DSC 0.97, interquartile range [IQR] 0.91, 0.99]). The model demonstrated similar performance with both fibreoptic scopes (0.97 [IQR 0.91, 0.99]) and digital scopes (0.97 [IQR 0.92, 0.99]; Fig. 1). The model also demonstrated good performance during stone localisation (0.98 [IQR 0.93, 0.99]) and stone laser ablation (0.96 [IQR 0.89, 0.97]), with a slightly worse performance during evaluation of residual fragments (0.91 [IQR 0.50, 0.97], Fig. 2). The model maintained real‐time performance at 30 FPS (Video S1).

### Model Comparison to Experts

Of the 200 frames for which model performance was compared to that of experts, 73 did not contain a stone. The model correctly identified 123 (97%) of 127 frames with a stone and 69 (95%) of 73 frames without a stone. The true‐negative and true‐positive rates achieved by the five experts ranged from 0.88 to 0.97 and 0.90 to 0.98, respectively (Table 1). The model's overall accuracy in identifying stone presence was 0.96 (95% CI 0.92–0.98), with only one expert surpassing the model (Expert 4: 0.97, 95% CI 0.94–0.99).

**Table 1:** Comparison of model performance (95% CI) to that of the five experts for identifying presence or absence of stone in each frame.

	True negative (*N* = 73)	True positive (*N* = 127)	Overall accuracy (*N* = 200)
Model	0.95 (0.87, 0.99)	0.97 (0.92, 0.99)	0.96 (0.92, 0.98)
Expert 1	0.88 (0.78, 0.94)	0.98 (0.94, 1.00)	0.95 (0.90, 0.97)
Expert 2	0.96 (0.89, 0.99)	0.90 (0.83, 0.94)	0.92 (0.87, 0.95)
Expert 3	0.96 (0.89, 0.99)	0.96 (0.91, 0.99)	0.96 (0.92, 0.98)
Expert 4	0.97 (0.91, 1.00)	0.97 (0.92, 0.99)	0.97 (0.94, 0.99)
Expert 5	0.88 (0.78, 0.94)	0.98 (0.93, 1.00)	0.94 (0.90, 0.97)

**Fig. 3:**
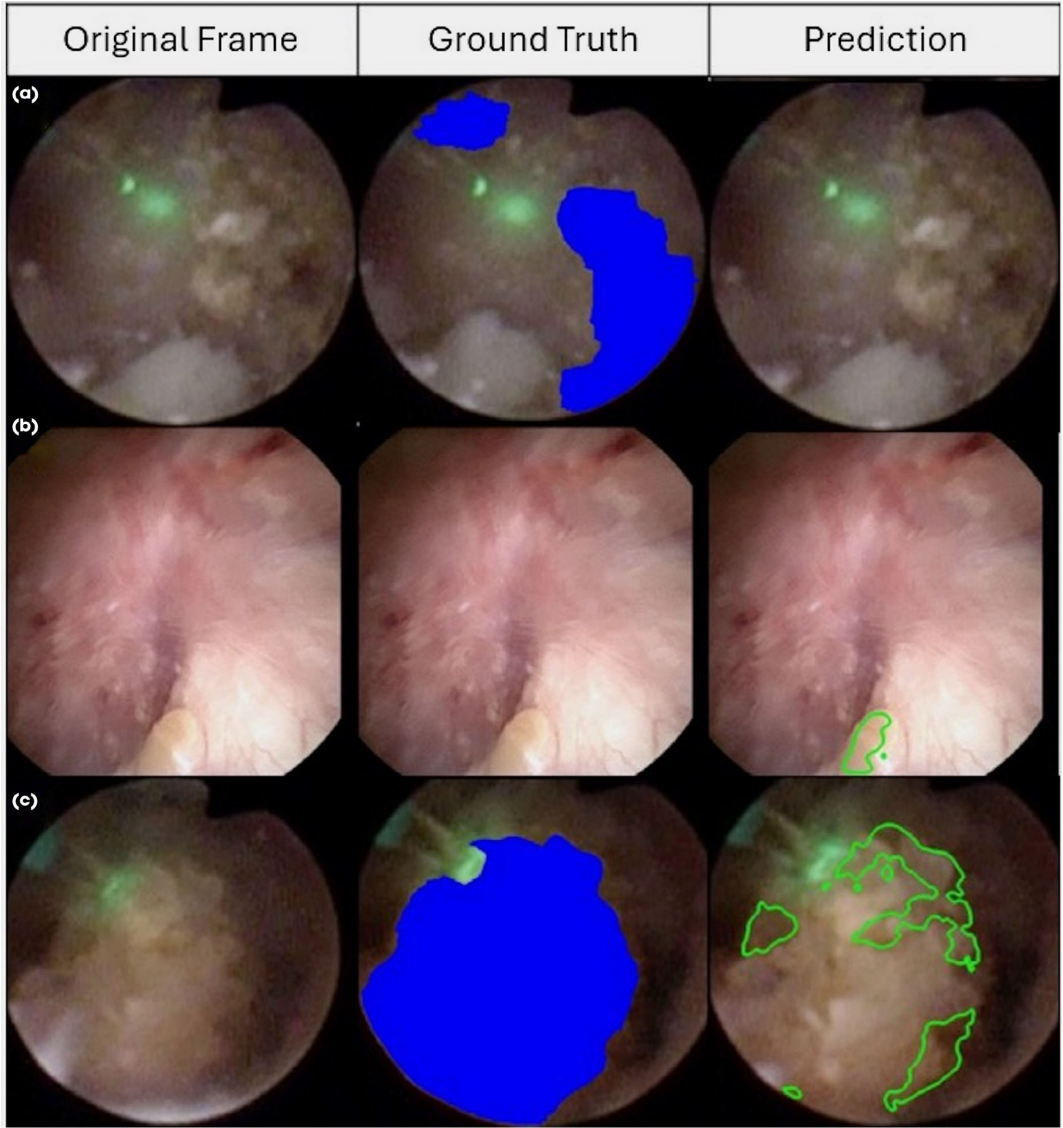
Example frames in which there was discordant stone prediction by the model compared to the ground truth. The left column shows the original, unannotated frames. The middle column displays the ground truth via manual annotation of stones represented by the blue overlay in the images. The right column demonstrates model prediction with the green outline overlay displaying the model prediction of stone. (**a**) Demonstrates a frame where the model did not identify stone when present, (**b**) demonstrates the model identifying stone when absent, and (**c**) represents the model only partially identifying stone when present.

The DSC was computed on the 127 frames with a stone, and head‐to‐head comparisons were performed on these frames for the model and each expert. The median DSC for the model was 0.94 (IQR 0.87, 0.97), which was comparable to the five experts (Table 2). Additionally, the winning proportion (i.e., the proportion of frames where the model identified stones more accurately based on DSC) was significantly higher than that achieved by three of the five experts (Table 3). The model only demonstrated stone prediction discordant from every expert in three frames. One of these frames was from a video of stone localisation, and two of these frames were from videos of stone ablation. Examples of discordant stone prediction are depicted in Fig. 3.

**Table 2:** Comparison of segmentation performance between the model and experts based on Dice similarity coefficient.

	Failure to identify stone when present (%)	Observed median DSC (Q1, Q3)
Model	4 (3)	0.95 (0.92, 0.97)
Expert 1	2 (2)	0.94 (0.91, 0.96)
Expert 2	13 (10)	0.92 (0.89, 0.96)
Expert 3	5 (4)	0.93 (0.89, 0.97)
Expert 4	4 (3)	0.93 (0.89, 0.97)
Expert 5	3 (2)	0.91 (0.88, 0.97)

DSC, Dice similarity coefficient. When the model or expert failed to identify a stone, the DSC was set to zero.

**Table 3:** Head‐to‐head comparisons of model win proportion for kidney stone segmentation.

	vs Expert 1	vs Expert 2	vs Expert 3	vs Expert 4	vs Expert 5
Frames	127	126	127	127	127
Model winning proportion (95% CI)	0.58 (0.49, 0.67)	0.68 (0.59, 0.76)	0.69 (0.60, 0.77)	0.57 (0.48, 0.65)	0.68 (0.59, 0.76)
*P* value	0.21	<0.001	0.002	0.06	<0.001

## Discussion

We present a computer vision model that accurately segments stones from URS performed with both digital and fibreoptic scopes. The model demonstrated good performance during specific surgical tasks of URS and versatility throughout the surgery. Moreover, the model showed similar segmentation performance when compared to board‐certified urologists. The model segments at 30 FPS, thereby enabling real‐time annotation of kidney stone video feeds for intra‐operative application.

We have previously evaluated preliminary models for automatic stone segmentation during URS [5, 6]. Although the models demonstrated good performance for overall stone segmentation, they were trained across a limited, homogenous dataset of 20 surgical videos from digital scopes depicting only stone localisation. As a result, the models’ performance in stone segmentation with fibreoptic scopes or during active stone treatment was limited. Previous studies have evaluated similar models using different computer vision techniques, but in those studies the datasets used for training were also small [7]. Gupta et al. [7], for example, compared several models for endoscopic stone segmentation in 440 frames, achieving a DSC of 0.82. Similarly, Leng et al. [12] developed a segmentation model with the intent of predicting stone composition during stone localisation in 50 videos (with only 1677 extracted frames), with a DSC of 0.91. Regardless of the computer vision architecture being implemented, model performance is significantly impacted by the size and type of data in the dataset.

In the current study, our model was trained on a heterogeneous dataset from 136 surgical videos (21 718 frames) that included both fibreoptic and digital scopes, as well as examples of the specific surgical tasks of URS. Thus, the model is much more robust for accurate stone segmentation across a variety of clinical scenarios and can be used throughout URS. We did see a decrease in performance when evaluating residual stone fragments (i.e., dust). This is likely due to both a smaller number of videos for evaluating residual stone fragments and the fact that these are smaller fragments which could be obscured by debris after stone treatment. These fragments can be difficult to track even by experts as no single surgeon successfully identified every stone fragment when visualising individual frames (Table 2). As these fragments could contribute to recurrence events, it is essential that we improve performance in these cases. In the future, we plan to leverage the temporal context from endoscopic videos (i.e., via bootstrapping later frames in a video clip containing stone fragments with the data from earlier frames) to improve the performance of our model for identifying smaller fragments. Additionally, we plan to implement a transformer model to capture contextual relationships across the video [13].

In this study, we compared model performance against the segmentations of five board‐certified urologists. The model performed similarly to the experts overall and outperformed three of the experts in a head‐to‐head comparison. This confirms the model's ability to accurately visualise stones during URS. The differences observed in expert performance highlight the inter‐operator variability in stone identification. Understanding inter‐operator variability among surgeons is essential for establishing a benchmark to compare machine learning‐based surgical tools. In this instance, the differences in performance among the surgeons likely reflect each different surgeon's level of experience in endoscopic stone surgery. Surgeons with more experience in treating kidney stones are better at stone identification and tracking during URS. This has been demonstrated in prior studies associating kidney stone‐related surgical outcomes with surgeon experience [14, 15]. It is possible that real‐time integration of the model could augment the visibility for less experienced surgeons.

In addition to potentially improving stone visualisation, our model could be used to objectively analyse endoscopic stone surgery. For example, we have demonstrated the potential for leveraging similar computer vision models to determine metrics that distinguish surgical expertise or stone‐free outcomes [16, 17]. By accurately integrating the stone segmentation model in future computer vision tasks, we could further identify surgical factors that might reduce operating times, improve stone‐free rates, and reduce stone‐related complications. Moreover, the model is software‐based so it could be integrated into any endoscopic system for stone treatment, making it widely usable.

This study has some limitations. First, all surgical videos were from a single institution and were of surgeries performed by five surgeons with only digital and fibreoptic Storz ureteroscopes. Despite the large dataset, which included examples that depicted the variety of endoscopic scenarios seen during URS, the model still requires external validation. We plan further evaluation of the model at other institutions with different surgeons and scope types. Additionally, we used holmium laser lithotripsy to evaluate the computer vision model during stone treatment. Although we do not anticipate laser modality to impact overall model performance, further evaluation of model performance using different laser modalities is needed. Furthermore, in certain situations, the frame rates of modern endoscopes may not sufficiently track rapidly moving stone fragments. As ureteroscope technology evolves, with improved resolution and frame rates, our model could be further refined to improve accuracy in some of these situations. Although the DSC accounts for both false positives and false negatives, it does not provide an explicit breakdown of these error types. Reducing false positives is important, as over‐segmentation may lead to indication of a stone when it is not present. Future work will focus on further evaluating our model to better understand and minimise error in stone segmentation. Additionally, the models were trained and implemented using an RTX A6000 Ada (NVIDIA) graphic processing unit. Validation of the model on other systems and an assessment of computational requirements is required.

Future work is needed to further improve the robustness of our model for tracking residual stone fragments and other tasks during URS. Additionally, model evaluation using other systems for broad, real‐world integration is required. We plan to conduct future user studies to ensure effective design for clinical use. This would involve comparing different overlay appearances (e.g., complete overlay vs outline) to assess how they may impact endoscopic visibility. We also plan to optimise the model for situations where visibility is limited by debris or bleeding to better guide surgeons for stone treatment intra‐operatively. Additionally, we seek to evaluate the impact of the model on automated surgical robotic systems.

In conclusion, we developed and evaluated a computer vision model for the automatic segmentation of kidney stones during URS in real time. The model demonstrated good performance for both fibreoptic and digital scopes. Additionally, the model demonstrated accurate stone segmentation during specific surgical tasks in URS. We found that the model performed similarly to a group of experts, which demonstrates the feasibility of its clinical use.

## Disclosure of Interests

This was a collaborative investigator‐led project. Drs Nicholas Kavoussi, Ipek Oguz, Tatsuki Koyama, and David Lu were partly supported by the National Institute of Diabetes and Digestive and Kidney Diseases through the National Institute of Health. Ekamjit Deol has no disclosures of interest.

## Funding

NIH/NIDDK (R21DK133742).

## Supporting information


**Video S1.** Demonstration of automated kidney stone segmentation during ureteroscopy.

**Table S1.** Demographic and video information for data set.
**Table S2.** Performance of different model architectures on the test cohort.

## Data Availability

Segmentation models can be found at the following link: https://github.com/MedICL‐VU/Kidney‐Stone‐Segmentation. The datasets analysed during this study are available from the corresponding author on reasonable request.
