# Supplementary material for: A computer vision model for automated kidney stone segmentation and evaluation of its performance vs surgeons
Source: BJU Int. 2025 Sep 25;137(1):87–94. doi: 10.1111/bju.70001 (PMC12690337; doi:10.1111/bju.70001)
Supplement: Supplementary file 2 — Table S1. Demographic and video information for data set. Table S2. Performance of different model architectures on the test cohort. [file BJU-137-87-s002.docx]

Supplementary table 1. Demographic and video information for data set

|  | Total Videos  N=136  (21,718 frames ) |
| --- | --- |
| **Demographics** |  |
| Age (years, mean±SD) | 53+-14 |
| Body mass index (mean±SD) | 31+-7 |
| Gender, male (N,%) | 63 (46) |
| Gender, female (N,%) | 73 (54) |
| Race, Caucasian, (N,%) | 118 (88) |
| Race, African American, (N,%) | 9 (6) |
| Ethnicity, Hispanic (N,%) | 9 (6) |
| **Endoscopic Video type** |  |
| Digital ureteroscope (N,%) | 98 (72) |
| Fiberoptic ureteroscope (N,%) | 38 (28) |
| **Depicted surgical task in videos** |  |
| Stone localization (N,%) | 67 (49) |
| Stone laser ablation (N,%) | 92 (67) |
| Evaluation of residual fragments (N,%) | 24 (18) |

Supplementary Table 2: Performance of different model architectures on the test cohort

|  | Dice Similarity Coefficient on Test Cohort | AUC-ROC on Test Cohort | Binary Cross Entropy Loss on Test Cohort |
| --- | --- | --- | --- |
| U-Net | 0.85 ± 0.07 | 0.98 ± 0.01 | 0.18 ± 0.11 |
| U-Net ++ | 0.84 ± 0.07 | 0.99 ± 0.02 | 0.15 ± 0.10 |
| U-Next | 0.79 ± 0.09 | 0.98 ± 0.02 | 0.19 ± 0.14 |
